# Natural Fibre as Reinforcement for Vintage Wood

**DOI:** 10.3390/ma13214799

**Published:** 2020-10-27

**Authors:** Agnieszka Wdowiak-Postulak

**Affiliations:** Faculty of Civil Engineering and Architecture, Kielce University of Technology, 25-314 Kielce, Poland; awdowiak@tu.kielce.pl; Tel.: +48-41-34-24-480

**Keywords:** vintage wood, reinforcing, natural fibres, flexural strength

## Abstract

In recent years, we have seen the construction of numerous good-looking buildings, each of which is perfectly safe, resistant to weather conditions, durable and economically efficient. Apart from their use in the structures of new buildings, natural fibres are even more important in the field of restoring historical heritage. The article presents experimental testing of old wooden beams made of the European larch *Larix decidua* Mill. with natural defects (knots, natural grain, deviations, cracks and voids) on a technical scale, reinforced with natural fibre. The tests were carried out to examine the response of heterogeneous wooden beams during bending with reinforced basalt fibres (BFRP). The wooden beams were cut out from the ceiling of an old building from 1860. The tests of reinforced wooden beams were intended to determine the increase in bearing capacity and rigidity after providing natural reinforcement. The tests allowed for determining the deflection, the distribution of deformations and images of failure for non-reinforced and reinforced beams. The performed tests have shown the effectiveness of the application of basalt fibres (BFRP) to the improvement of structural properties of existing beams, thus allowing an increase in flexural strength. It can be concluded that reinforcements using BFRP materials can be applied both to strengthening the existing structures with deteriorated mechanical properties, as well as to reduce the dimensions of a structure. Experimental tests have proven that, in the case of beams reinforced with natural basalt (BFRP), their rigidity increases by ca. 15.17% compared to the reference beams.

## 1. Introduction

Wood is the oldest construction material used by mankind. In the 20th century, steel and concrete began to replace wood as the main construction material used in the building industry. On the other hand, in the 21st century, wood is still used by many builders, primarily in the USA, to build short-span bridges [1,2]. Wood is also used for the construction of—e.g., residential buildings, commercial facilities and sports centers. Currently, numerous countries also use wood for the construction of multistory structures (such as, the Mjøstårnet project made utilising the most recent cross-laminated wood technology (CLT) [3]). It should be noted that wood has considerable strength at a low mass ratio; therefore, it is easy to erect and no shuttering is required.

Moreover, wood reduces building costs, since there is no need to use heavy machinery during its application. One should also note that the time required for the construction of wooden structures can be reduced, because, unlike in concrete structures, no extra time is needed for hardening. Moreover, the advantages of wood, especially in lightweight structures, include resistance to corrosion and acoustic and electrical insulation. Certain serious problems of wooden structures include the huge and growing consumption of wood products; however, wood is characterised by a long growth cycle and slow regeneration; unfortunately, there is still a considerable deficiency of its supplies [4,5,6].

Wooden structures are also subject to problems related to design errors, overload and numerous pests occupying wood and destroying it. Some design problems include the variable strength of wood and its water content. When designing wooden structures, it should be kept in mind that wood originating from the same log will have varying mechanical properties. Therefore, its strength can differ due to the varying orientation of grain. The reduction in the strength of the wood causes the grain direction to deviate from the direction parallel to the longitudinal axis of the trunk. The decrease in strength increases with increasing deflection angle. The highest compressive strength is found in wood, where the force acts parallel to the course of the fibres (longitudinal compression). In the tangential and radial direction (transverse compression) the strength is much lower. The tensile strength along the grain is the highest among the mechanical properties of wood. Depending on the type of wood, the tensile strength across the grain is approximately 5–40 times lower than the tensile strength along the grain. The highest bending strength results are shown by wooden beams in which the fibres run parallel to their edges. Wood without defects has greater strength than wood with defects. The number, size and quality of knots have a great influence on the strength of the wood. All these problems affect the bearing capacity of wooden structures. Therefore, wood reinforcement methods should be expanded in order to increase the bearing capacity of wooden structures [1]. There are many options of reinforcing wooden elements, used both in new structures and in the renovation of existing objects.

Studies on methods used to reinforce wooden structures are relatively vast, and it can be concluded that they have been performed properly [7,8,9,10,11,12,13,14,15,16,17,18,19,20,21]. Researchers Li et al. [22] described the use of a CFRP sheet in a tension zone for two different wooden beam types. In their studies, they used various layers of the FRP material. During the tests, researchers Li et al. [22] determined the displacement–load ratio. After completed studies, they concluded that the average deflection of beams decreases with growing layers of CFRP sheets. The increase in flexural strength for Cunninghamia lanceolata with 1, 2 and 3 layers of CFRP sheets was 39%, 44% and 61%, respectively. The increase in bending strength of Tsuga chinensis with 1, 2 and 3 layers of CFRP sheets was 44%, 55% and 58%, respectively. The studies of Borri and Corradi [23,24] show that flexural strength of old wooden beams increases within a range of 30% relative to non-reinforced beams when using reinforcement, based on CFRP and GFRP composite materials. After the performed tests, it was concluded that very interesting efficiency of reinforcement can be achieved when using external FRP reinforcement. In the case of three layers made of carbon fibres, flexural strength increased by up to 60.3%, compared to non-reinforced beams. Strength tests were also performed for the reinforcement of solid wooden beams with composite materials using carbon fibre fabrics (CFRP), [25,26,27]. The tests used the cross-sections of wooden beams along with existing cracks, knots and damage, reducing the initial flexural rigidity and moment of inertia by approximately 17%, respectively, compared to beam cross-sections without defects. Wooden beams reinforced with CFRP exhibited more plastic properties compared to non-reinforced beams. Based on the tests, it was concluded that strengthening based on the utilisation of CFRP reinforcement limits local fractures and defects in wood. Failure occurred due to shear caused by the splitting of the cross-section in a lengthwise direction between the occurring fractures, with a load level between 35 kN and 87 kN (an average of 53.16 kN). The tests indicated that the most frequent damage mechanism is the one in which damage to traction and failure by shear occurs with or without partial yielding of the compressed zone. The use of reinforcement in wooden beams increased the bearing capacity by an average of 5.86% compared to non-reinforced wooden elements. The authors in [28] tested old, 100 years old beams under a four-point flexural load. The distributions of deformations were determined using resistance strain gauges (ERSG) along with the photoelastic coating technique (PCT). Based on the tests, it was concluded that the values of deformations measured by means of ERSG and PCT exhibited considerable differences (of 2.2–72.5%) related to the significant impact of wood structure on local deformation patterns. The results of deformation measurements proved that the proposed methods of reinforcing wooden beams by means of CFRP are advantageous for the purposes of renovation. More bending tests of wooden elements reinforced with composite materials were performed by researchers Fiorelli et al. [29]. In these tests, experimental values were close to theoretical ones. The increase in rigidity ranged from 15 to 30% in beams reinforced with 1.0% fibreglass and 0.4% carbon fibre. In the case of a beam reinforced with 3.0%, fibreglass rigidity increased by approximately 60%. Additionally, Borri et al. [23] achieved an increase in the bearing capacity of existing wooden beams when using CFRP, GFRP and AFRP rods and belts. Researchers Borri and Corradi [30] also determined the effectiveness of flexural reinforcement made using steel fibres embedded into the tension zone of wooden beams. In certain wooden elements, the increase in maximum load exceeded even 100% compared to non-reinforced beams. Researchers Gentile et al. [31] carried out bending tests for side sections of wooden beams reinforced with GFRP rods. Alam et al. [32] reinforced cracked wooden beams with steel and the FRP material. The results proved that these reinforcements effectively improved the flexural strength of wooden elements. Reinforcements embedded simultaneously into the upper and lower surfaces of the beams were more effective than those embedded only into its upper or lower surface. Wood is characterised by low plasticity; therefore, deflections increase under failure load until cracking, when the splitting of fibres occurs. In this phase of destruction, the rod loses its bearing capacity. This was observed during tests performed by Gardner [33].

It should be noted that many reinforcement studies were carried out for glulam beams. On the other hand, the number of studies involving the reinforcement of solid wood is still limited. The subject literature includes experimental studies carried out using materials reinforced with artificial fibres (e.g., glass, carbon, aramid fibres, etc.) based on epoxy resins. Indeed, these studies have proven that the use of these composite materials is of considerable structural importance, because they ensure higher strength and plasticity of wooden elements compared to the properties of non-reinforced wood. On the other hand, there is still absence of experimental studies involving vintage wood reinforced with natural fibre, such as basalt, bamboo, cotton, flax, hemp, etc. This is why the paper describes experimental tests of wooden beams reinforced to the inside in the tension zone with natural fibre (BFRP) on a technical scale. This study will focus on the response of reinforced and non-reinforced heterogeneous vintage wood during four-point bending. This will help with the design of new buildings and the renovation of existing wooden structures. One should keep in mind that the rehabilitation and reinforcement of existing wooden structures will improve their bearing capacity, which may save the costs of replacement with a new wooden structure. Additionally, efficient reinforcement techniques may also reduce the sizes or spans of wooden beams required for their construction, as was observed in the studies of Haiman and Zagar [34], at the same time increasing their strength, resulting in the efficient use of wood supplies. These tests were performed for actual samples of glulam beams spanning 3.9 m, their objective being to determine the possibilities of reinforcing the beams with FRP boards. It is known that a larger cross-sectional area of wooden beams is hard to obtain, while a small cross-sectional area of wooden beams after reinforcement may provide mechanical properties equivalent to those of the larger one. Besides, the following experimental studies will determine whether the existing structures with considerably reduced mechanical properties can still work in existing buildings after being reinforced.

## 2. Materials and Methods

### 2.1. Test Materials

#### 2.1.1. Wood

The main test material in these experimental studies constituted heterogeneous wooden beams of the European larch *Larix decidua* Mill., originating from an antique building from 1860. Since it is not possible to share their exact origins, this information is not included in here. The final size of solid beams was 82 mm × 162 mm × 3650 mm. After cutting the beams out from the antique building, their structural and geometrical features were characterised, followed by their categorization into specific sorting classes according to strength sorting carried out using the visual method [35]. Apart from that, the measured parameters included the average width of rings, water content, mass and density of wood samples under air-dry conditions [36,37].

All wooden beams reached their preferred water content of 12%. This is extremely important, as it allows for ignoring the relationship between water content and flexural strength. The following parameters were determined during strength sorting using the visual method: the knottiness index of the marginal zone (USM)—an index covering one of the two marginal zones, meaning one in which the knots occupy a larger space, the so-called worse margin; the general knottiness index (USC)—a knottiness index referring to the whole cross-sectional area of lumber. The visual sorting is always burdened with the “human factor”, so the sorting result depends on the sorting person. The error range in this study was 1.5%. After the completion of this visual analysis, it was concluded that the tested material belongs to the worst sorting group, KG—lower quality class. Structural and geometric features of sample wooden beams (the location of the bases is shown in Table 1):

(1) Beam NW_BFRP_-1

USM = 0.38, USC = 0.25, grain—7.2 mm, the twist of the fibres—1:7, density—424.15 kg/m^3^. **Front:** heart shake (length 41 mm, depth 4 mm), twist (1.2 mm), longitudinal bowing of sides (10.2 mm), oval knot (partly spoilt)—bases 1, 10; longitudinal knot (partly spoilt)—bases 2, 3; oval knot (sound)—bases 3, 6; longitudinal knot on the edge—base 3. **Back:** blue stain, nail traces, oval knot (spoilt)—bases 2, 6, 11, 13. **Bottom:** oval knot (partly spoilt), oval knot (sound)—base 4; oval knot (spoilt)—base 9. **Top:** oval knot (partly spoilt)—bases 2 and 3.

(2) Beam NW_BFRP_-2

USM = 0.42, USC = 0.35, grain—7.4 mm, the twist of the fibres—1:8, density—429.45 kg/m^3^. **Front:** twist (1.4 mm), longitudinal bowing of sides (11.5 mm), heart shakes (length 37 mm, depth 3.4 mm), oval knot (spoilt)—bases 1, 4, 12; oval knot (partly spoilt)—bases 4, 8, 11, 12; longitudinal oval knot—base 8; decay (length 1820 mm, depth 1 mm)—bases 11 and 12. **Back:** oval knot (partly spoilt)—bases 4, 8, 12. **Bottom:** oval knot (partly spoilt)—bases 3, 8, 12, 10. **Top:** oval knot (partly spoilt)—bases 4, 8, 12.

(3) Beam NW_BFRP_-3

USM = 0.46, USC = 0.39, grain—7.1 mm, the twist of the fibres—1:6, density—418.25 kg/m^3^. **Front:** nail traces, blue stain, oval knot (partly spoilt)—bases 1, 4, 11; oval knot (spoilt)—bases 1, 7, 8; oval knot (partly spoilt) on the edge—base 4. **Back:** longitudinal knot (sound) on the edge—bases 2, 6, 8; oval knot (sound)—bases 7, 8, 12; oval knot (spoilt), longitudinal knot on the edge—base 12. **Bottom:** oval knot (partly spoilt). **Top:** oval knot (partly spoilt).

(4) Beam W_BFRP_-1

USM = 0.52, USC = 0.31, grain—6.7 mm, the twist of the fibres—1:7, density—423.48 kg/m^3^. **Front:** nail traces, heart shake (length 33 mm, depth 3.1 mm), longitudinal bowing of sides (11.9 mm), twist (1.6 mm), oval knot (sound)—bases 2, 4, 6, 9, 10; longitudinal knot on the edge—base 6; oval knot (partly spoilt)—bases 8, 11, 12, 13. **Back:** wane (53 mm, 22 mm), non-penetrating cracks (length 128 mm, depth 5.6 mm), oval knot (spoilt)—bases 2, 6; oval knot (sound)—bases 4, 6, 8, 9, 10, 11, 13; bark pocket (length 42 mm, depth 3.3 mm)—base 4; oval knot (partly spoilt)—base 12. **Bottom:** oval knot (sound)—bases 2, 4, 8, 10; non-penetrating crack (length 115 mm, depth 5.1 mm). **Top:** oval knot (sound)—base 2.

(5) Beam W_BFRP_-2

USM = 0.37, USC = 0.34, grain—7.9 mm, the twist of the fibres—1:6, density—411.48 kg/m^3^. **Front:** longitudinal bowing of sides (10.5 mm), twist (1.4 mm), blue stain, non-penetrating cracks (length 97 mm, depth 4.2 mm), insect runs, oval knot (spoilt)—base 3; longitudinal knot (partly spoilt) on the edge—base 3; oval knot (partly spoilt)—bases 6, 10. **Back:** longitudinal knot—base 1; oval knot (sound)—bases 2, 5, 6, 7, 10, 11; longitudinal knot on the edge—bases 2, 3, 4, 5, 7, 10; oval knot (sound) on the edge—base 11.

(6) Beam W_BFRP_-3

USM = 0.31, USC = 0.29, grain—8.2 mm, the twist of the fibres—1:6, density—404.92 kg/m^3^. **Front:** longitudinal bowing of sides (11.9 mm), twist (1.8 mm), blue stain, non-penetrating cracks (length 156 mm, depth 5.2 mm), nail traces, oval knot (spoilt) - base 1; oval knot (sound)—bases 2, 10, 13; oval knot (spoilt) on the edge—base 2; oval knot (partly spoilt)—bases 4, 5, 7; oval knot (partly spoilt) on the edge—base 7. **Back:** non-penetrating cracks (length 85 mm, depth 3.2 mm), oval knot (spoilt) on the edge—bases 1, 5; oval knot (spoilt)—bases 1, 3, 4, 5; oval knot (partly spoilt)—bases 7, 10, 12; wane—base 11; oval knot (partly spoilt) on the edge—base 12. **Bottom:** partly spoilt knot—base 6; non-penetrating cracks (length 137 mm, depth 5.5 mm). The drawings of the test samples are shown in Table 1.

#### 2.1.2. Epoxy Adhesive

Epoxy resin-based adhesives bond material composites due to their excellent gap filling properties and reduced contractions while setting. Epoxy adhesives can set completely at an ambient temperature and they do not need to be pressed strongly. It should be kept in mind that an important task of adhesives used in composites involves the sufficient distribution of stress among fibres, which protects the fibres against corrosion and friction.

The epoxy adhesive produced by GRM Systems (LG815 + HG353) used in the experimental tests is a structural binary adhesive with the following properties: flexural strength of 110–120 MPa and elastic modulus of 2700–3300 MPa. The epoxy adhesive (LG815 + HG353) consisted of two components mixed in a ratio of 2.5:1 (by volume), according to product standard requirements. The epoxy resin-based adhesive layer was generated by mixing LG 815 epoxy resin (density 1.13–1.17 g/cm^3^, viscosity 1100—1300 mPa·s) with HG 353 hardener (density 0.98 g/cm^3^, viscosity 100–150 mPa·s).

#### 2.1.3. The BFRP Basalt Fibre

It can be observed that the FRP composite material is produced by mixing a fibre material and a matrix (epoxy resin) based on a predetermined proportion. Therefore, the fibre constitutes a strengthening material, which improves both the bearing capacity and rigidity of the composite, while the matrix distributes load among fibres. 

Basalt fibres form a unary material obtained by melting solidified volcano lava. These fibres have better physicomechanical properties than glass fibres and they are relatively inexpensive. As a result of this, these fibres, apart from E-glass fibres, obviously, are the most preferred materials for the production of composite rods. The primary advantages of basalt fibres include: fire resistance, high acoustic insulating power, internal vibration damping capacity, high fatigue strength, fibre operation temperature (982 °C), fibre melting temperature (1450 °C), high hardness, corrosion resistance.

Compared to steel rods, basalt composite rods have more than doubled tensile strength [BFRP (up to 1450 MPa), steel (500–600 MPa)]; they are four times lighter and twice more durable, resistant to corrosion, acids and bases; they have dielectric properties and are transparent to electromagnetic waves, also being cheaper than steel rods.

The tests included the making of reinforcements by applying an epoxy adhesive and prestressed the BFRP basalt fibres. The values of elastic moduli and the final deformations of the BFRP amounted to E = 78 GPa and Ɛ_u_ = 39%, respectively.

### 2.2. Preparation of Reinforcement for Wooden Beams

**Figure 1:**
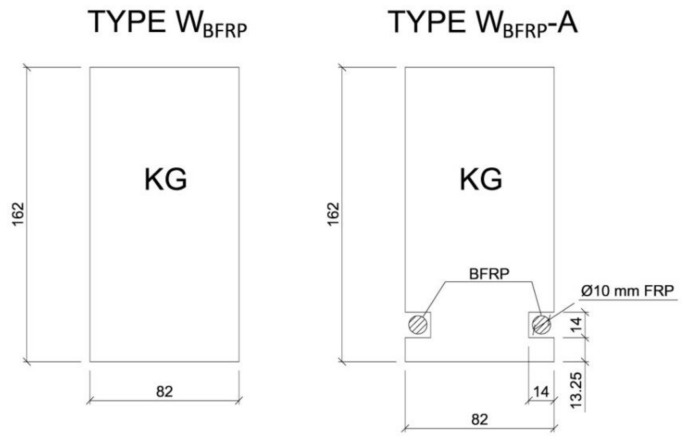
Cross-sections of the tested beams, type “W_BFRP_” (dimensions in mm).

Three beams were not reinforced and they served as reference beams (type NW_BFRP_). On the other hand, the remaining beams were reinforced using the BFRP basalt fibres (type W_BFRP_-A). Figure 1 shows the cross-sections of all beam types used in the experimental studies. Three repetitions were tested for each configuration to ensure reliability of the produced results.

The W_BFRP_-A type beams were reinforced with prestressed BFRP fibres, 10 mm in diameter. Each wooden beam was cut parallel to the fibres along its entire length to prepare two grooves along the bottom section of the beam. For better comparison, the groove sizes were the same in all beams—ca. 14 mm. The grooves were cleaned with a brush and an acetone solvent in order to remove any soiling. This phase is very important to ensure sufficient bonding between the BFRP fibres and wood. Subsequently, the BFRP fibres were cut to a length of 3.75 m. The first layer of the epoxy adhesive (LG 815, HG 353) was applied inside the grooves. The composite fibres was fixed and pre-stressed to approximately 40 MPa (5 mm—thick sheet metal, nuts) on the supports, which corresponds to a 3 mm elongation of the BFRP fibres along the beam’s length. The following layers of the epoxy adhesive (LG 815, HG 353) were then applied to fully cover the BFRP fibres. The descriptions of both beam types are provided in Table 2. All wooden beams were left for a minimum of seven days in a room with stabilized conditions of relative humidity 65 ± 5% and temperature 20 ± 2 °C until full setting, to ensure permanent bonding between the FRP and wood.

### 2.3. Experimental Tests

The research was aimed at improving the mechanical properties, as well as ensuring better reliability of this type of structural elements. During the tests, an experimental program was carried out to analyse the flexural strength of low quality wooden beams reinforced with natural fibre.

**Figure 2:**
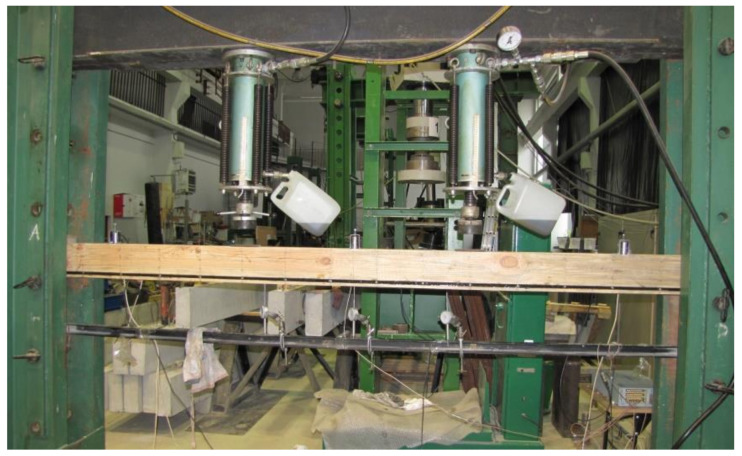
The experimental set-up scheme of a reinforced beam W_BFRP_-A2 (photo by: Wdowiak-Postulak).

The beams were tested at the laboratory of the Faculty of Civil Engineering and Architecture at the Kielce University of Technology. The test bench consisted of two actuators with a piston surface area of 50 cm^2^ and a maximum generated pressure of 10 MPa, manufactured by VEB Werkstoffprufmaschinen Leipzig (Markkleeberg, Germany). The static bending test was carried out according to quality standard PN-EN 408 + A1:2012 [38]. Figure 2 shows the experimental set-up scheme of a reinforced beam W_BFRP_-A2. A wooden beam was placed on the supports of a strength machine with measurement bases distributed over the entire length of the beam followed by the installation of mechanical sensors for measuring deflection. 

The load was applied. The wooden beams were subjected to loads starting from 0 kN, increasing the value of the loading force until reaching their complete destruction.

The test was carried out by reading:

(1) The value of the loading force;
(2) Beam displacement at mid-span and at a length of 5 *h* (where *h*—cross-sectional height of the beam);
(3) Deformations in wood;
(4) Deformations in natural fibres;
(5) The value of breaking force, also determining the manner of destruction of the tested beams.

First, the reference beams (type NW_BFRP_) were tested until their complete destruction, followed by the reinforced beams (type W_BFRP_).

The respective dimensions of wooden beams were 82 mm × 162 mm × 3650 mm. Static bending tests were performed for a beam height-to-span ratio of 18. The beams were of low quality, with typical wood defects, including lateral rings, contraction cracks and knots. Figure 2 shows the distance between supports and the distance between the applied concentrated forces. Beams supported freely on both ends were subjected to a symmetrical load, in two points by concentrated forces F/2.

**Figure 3:**
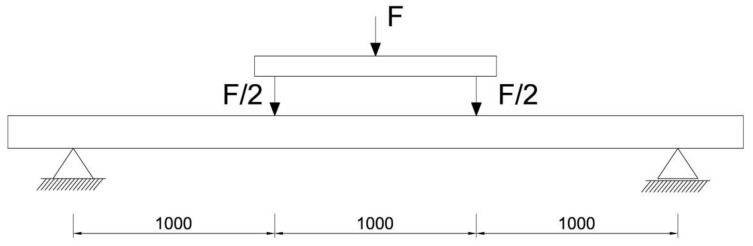
Static test of four-point bending (dimensions in mm).

All wooden beams gave up to the four-point load. The clear span of wooden beams amounted to 3000 mm (see Figure 3). 

**Figure 4:**
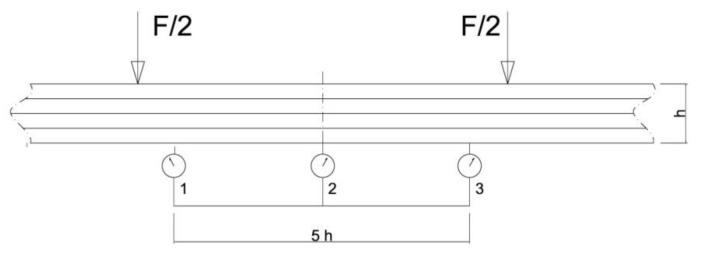
Layout of the distributed mechanical sensors.

Deflections of all beams were measured by three mechanical sensors placed under the tested beam. Figure 4 shows the layout of the distributed mechanical sensors. They were dial indicators with an accuracy of 0.01 mm.

**Figure 5:**
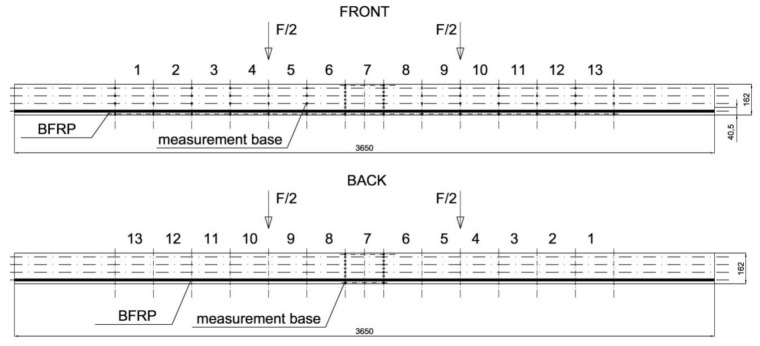
Layout of the measurement bases distributed at the front and at the back of W_BFRP_-A reinforced beams (dimensions in mm).

Besides this, the deformations of wood and natural fibres were measured during the tests using a mechanical extensometer with a fixed measurement base of the “Demeck” type, of 8 inches or 203.2 mm. The values of deformations were measured in each measurement base. Figure 5 shows the distribution of measurement bases. Over the entire length of the beam, its height was divided into four smaller fragments of 40.5 mm. Then, 13 measurement bases were established in each fragment at the half of its height. In the case of BFRP basalt fibres, the values of deformations were measured in 13 measurement bases over the entire beam length. All the measurement bases are shown in Figure 5.

## 3. Results

This study presents results related to the type of reinforcement of heterogeneous beams which use natural fibres. Composite materials (BFRP) are fitted inside the inner tension zone. The two different types of beams which were tested included NW_BFRP_ and W_BFRP_-A. The studies determined the flexural strength of wooden beams using a four-point bending test, as discussed in the following subsections: deflection characteristics, deformations and normal stresses of wood and natural fibres, along with the generated beam failure images. The performed tests resulted in an exceptional improvement of mechanical properties, including maximum flexural load and flexural rigidity related to the use of the BFRP basalt reinforcement. Moreover, the experimental studies have shown no defects in reinforcement bonding in all tested reinforced beams. The results shown below suggest that natural fibres are an excellent technology for the existing wooden structures with defects or design errors, or when used to reduce the dimensions of construction materials.

### 3.1. Deflection Characteristics

**Figure 6:**
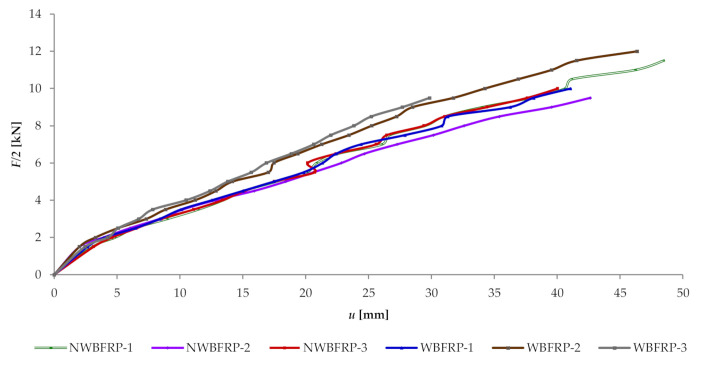
Graph of the “F/2–u” relationship in beams for sensor 2—mid-span of the beam.

The deflection–load curves (“F/2–u”), shown in Figure 6, present the range of rigidity for all tested beams.

It can be observed that reinforced beams initially work linearly, and with an increasing load they begin to work plastically in the compression zone. Failure occurred upon reaching the tensile stress limit amounting to 67 MPa in wood. The breakage was caused by the loss of adhesion between glue and wood with a stress of 240 MPa in BFRP fibres. It should be kept in mind that mechanical sensors were removed before the destruction phase.

The NW_BFRP_ beam types constituted reference beams relative to the W_BFRP_-A reinforced beams. For an F/2 of 5 kN, non-reinforced beams of the NW_BFRP_ type exhibited an average displacement of ca. 16.53 mm at beam mid-span, while for reinforced beams of the W_BFRP_-A type, after applying the BFRP basalt fibre, it amounted to 15.15 mm, respectively. The results are presented in Table 3. An increase in rigidity by ca. 9.11% was observed for beams reinforced with basalt fibre, compared to the non-reinforced beams.

### 3.2. Deformations and Normal Stresses

**Figure 7:**
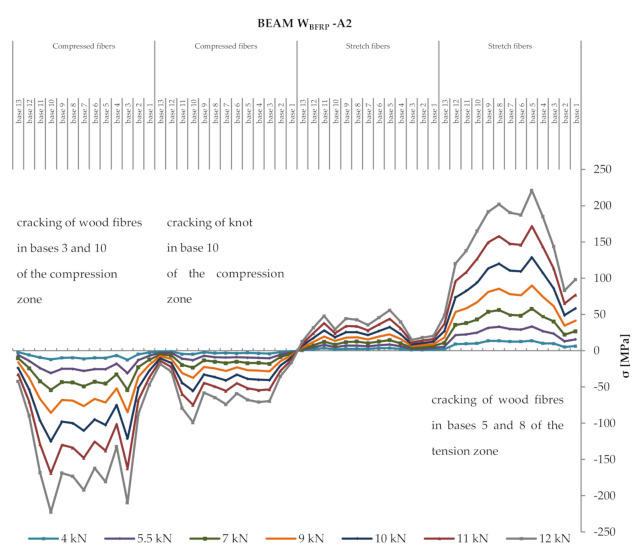
Distribution of normal stresses in wood *σ* (MPa) in the tension and compression zones of the cross-section over the entire length of a reinforced beam W_BFRP_-A2.

In the half of the length of each 40.5 mm fragment at the height of the wooden beam, 13 measurement bases (beam length—13 mm × 203.2 mm) were located, and then the deformation values were read using a mechanical extensometer at a given loading force F/2. To determine the normal stresses in wood, the modulus of elasticity of wood with the appropriate value of 11,300 MPa, calculated during experimental tests, was used. Figure 7 shows the distribution of normal stresses in the tension and compression zones of the cross-section over the entire length of a heterogeneous wooden beam reinforced with prestressed BFRP fibres.

Based on Figure 7, one can observe the uneven distribution of normal stresses. This results mainly from the occurrence of wood defects, usually cracking of knots, wood fibres, etc. In an occurring wood defect, natural fibres were taking over tensile forces, with an increase in stresses occurring therein. All natural defects are described in Figure 7 in order to show this effect of the impact of a heterogeneous wood structure on the efficiency of the applied BFRP reinforcement.

In the case of beam types of the lower quality class (W_BFRP_-A) reinforced with BFRP natural fibres at mid-span for an F/2 of 5 kN (base 7), it was observed that normal stresses increased in the tension zone of wood by ca. 17% compared to the NW_BFRP_ beam type. The detailed results of tensile and compressive stresses for the series of beams are presented in Table 4. The determination of normal stresses in wood used its elastic modulus on a technical scale, with a respective value of 11,300 MPa, calculated during experimental tests according to PN-EN 408 + A1:2012 [38].

**Figure 8:**
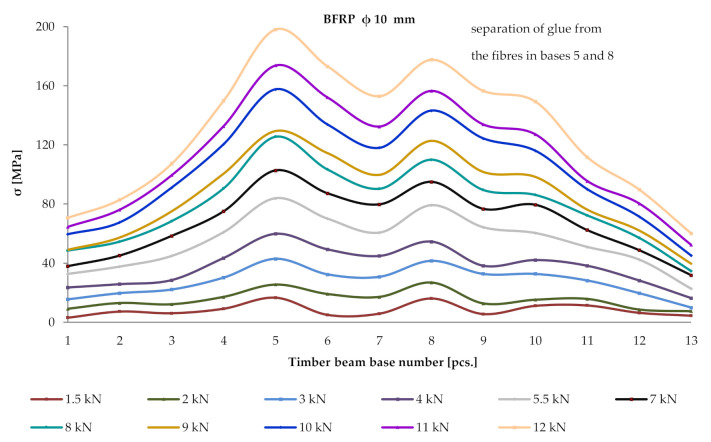
Distribution of normal stresses in the BFRP fibres *σ* (MPa) over the entire length of a reinforced beam W_BFRP_-A2.

Thirteen measurement bases (13 mm × 203.2 mm) were arranged along the length of BFRP fibres. For a given loading force F/2, deformation values were read in these databases using a mechanical extensometer. Then, the determination of normal stresses in BFRP fibres used the elastic modulus of BFRP with a respective value of 56,300 MPa, calculated during experimental tests. Figure 8 below shows the distribution of normal stresses over the entire length of a prestressed BFRP fibres. 

It can be observed that normal stresses within the reinforcement increased almost linearly until the moment of breakage. All wooden beams were destroyed by wood cracking and not by breaking of the BFRP reinforcement. Based on the performed tests, it has been concluded that the measured deformations in the reinforcement were smaller than the maximum tensile deformations.

### 3.3. Beam Failure Image

**Figure 9:**
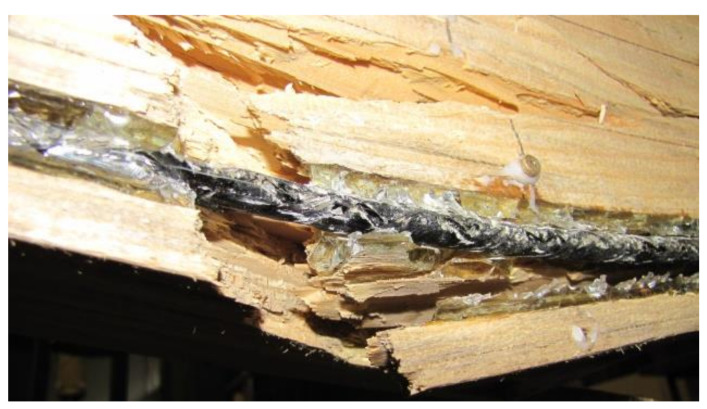
Sample image of the failure of a wooden beam reinforced with natural fibre (W_BFRP_-A2) (photo by: Wdowiak-Postulak).

In all types of beams, the breakage of shear reinforcement occurred due to a wood defect in the tension zone. It can be concluded that no BFRP reinforcement reached the maximum tensile stress. Considering the above, wood undergoes destruction in the tension zone as a result of its own defects—in most cases a knot or another defect (considerable degree of grain deviation). It should be noted that after providing reinforcement in the beam tension zone, the impact of wood defects is mitigated and the propagation of fractures is blocked. Figure 9 shows a sample image of the failure of a W_BFRP_-A2 beam. Tension and compression zones were destroyed under a load of 36 kN.

## 4. Conclusions

The following conclusions were derived on the basis of the experimental studies performed on a technical scale, involving vintage wooden beams of the European larch *Larix decidua* Mill. with natural defects (knots, natural grain, deviations, cracks and voids), reinforced with natural fibres:

(1) For an F/2 force of 5 kN, among the values of deflections for wooden beams, the highest one was exhibited by the series of reference beams NW_BFRP_-2. On the other hand, the lowest one was associated with the series of beams reinforced with the BFRP basalt fibre, with a reinforcement ratio of 1.17%—W_BFRP_-A3.
(2) At the mid-span of a beam for an F/2 force of 5 kN, the increase in tensile stresses of wood for a series of reinforced beams amounted to ca. 17% relative to non-reinforced beams. The highest values of tensile stresses of wood were exhibited by the series of W_BFRP_-A1 beams, the lowest ones by the series of non-reinforced beams NW_BFRP_-3.
(3) At the mid-span of a beam, for an F/2 force of 5 kN, the increase in compressive stresses of wood for a series of reinforced beams amounted to ca. 0.14% relative to non-reinforced beams. The highest values of compressive stresses of wood were exhibited by the series of W_BFRP_-A3 beams.
(4) At the mid-span of a beam, for an F/2 force of 5 kN, the average tensile stresses of BFRP fibres for a series of reinforced beams amounted to ca. 61.86 MPa. The highest values of tensile stresses of BFRP fibres were exhibited by the series of W_BFRP_-A1 beams.
(5) Beams combined with the prestressed BFRP basalt fibre subjected to submitted exhibited higher flexural strength compared to reference beams. This increase for a combined cross-section amounted to ca. 30%, relative to reference beams. The results of this experiment show that composite materials based on natural fibres allow for providing wooden beams with better strength and rigidity properties compared to non-reinforced wood, which makes it possible to use this natural reinforcement in the rehabilitation of antique wooden structures with deteriorated mechanical properties.
(6) In particular, beams reinforced in the tension zone with prestressed BFRP basalt fibres showed as much as a 36% increase in their bearing capacity compared to the non-reinforced beams. It can be cautiously stated that natural fibres caused a considerable increase in the bearing capacity of wooden beams, keeping in mind the limited statistical sample of the number of tested beams. Based on the performed experimental analysis, it was concluded that the reinforcement of lower quality beams with natural fibre resulted in an improvement in their bearing capacity.
(7) The completed performed experimental studies allowed a conclusion that for beams reinforced with the BFRP natural basalt, the increase in rigidity reached ca. 15.17% compared to the reference beams.
(8) In the tested beams, the failure of reinforcement occurred due to wood defects in the tension zone. Upon placement of the BFRP reinforcement in the tension zone of the beams, the impact of wood defects was mitigated and the propagation of fractures was blocked. At the moment of failure, some reinforced wooden beams exhibited plasticization of their cross-sections in the compression zone. Therefore, the reinforcement of beams with basalt fibres ensured good plastic properties. Moreover, based on the completed performed experimental analysis, it was also observed that the use of epoxy resin as a matrix for natural fibres may provide efficient bonding with wood.
(9) The use of natural fibre is a particularly prospective reinforcement technology, due to the lower costs of production, energy consumption and disposal. It should be noted that the technology of reinforcing prestressed natural BFRP fibres has a considerable potential for applications to new buildings and to the renovation of existing wooden structures with deteriorated mechanical properties.

## Figures and Tables

**Table 1:** Drawings with defects of sample wooden beams (NW_BFRP_-1, NW_BFRP_-2, NW_BFRP_-3, W_BFRP_-1, W_BFRP_-2, W_BFRP_-3—beam symbols).

Beam	Sample Picture
**NW_BFRP_-1**	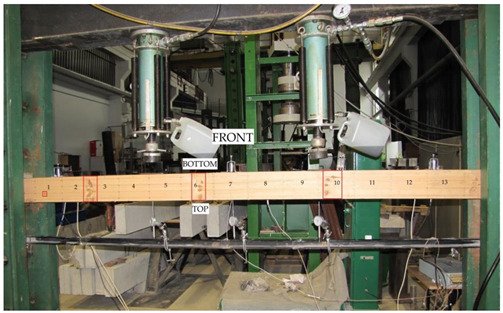
**NW_BFRP_-2**	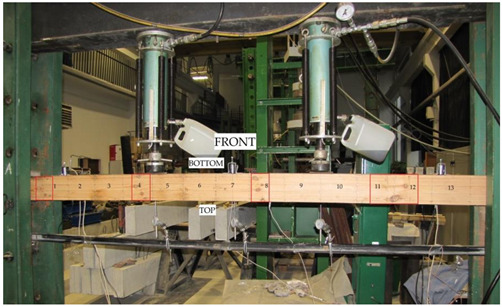
**NW_BFRP_-3**	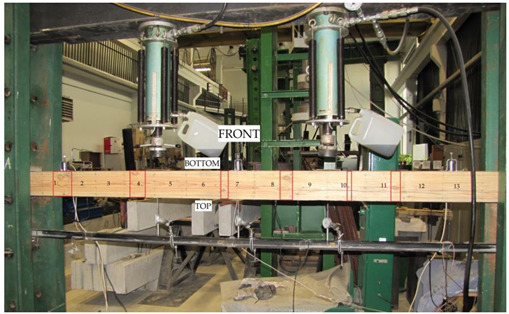
**W_BFRP_-1**	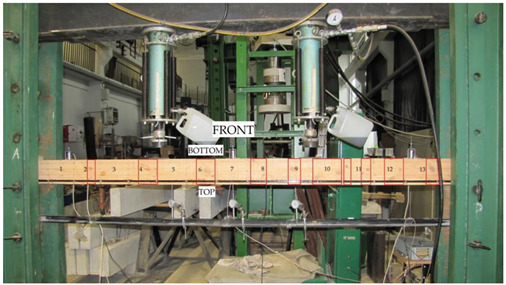
**W_BFRP_-2**	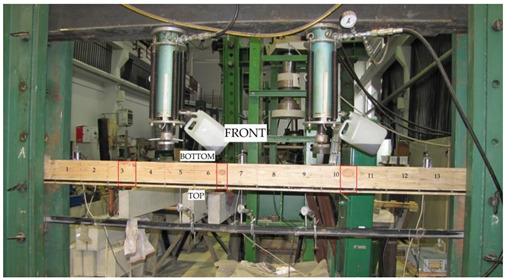
**W_BFRP_-3**	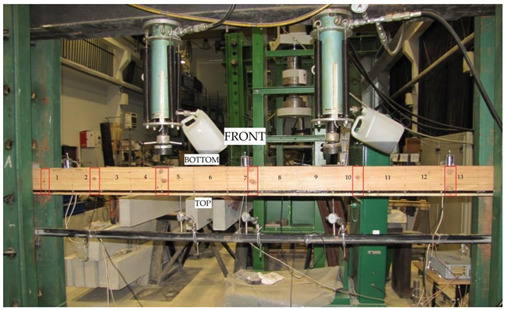

**Table 2:** Specifications of the types of old beams of the European larch, lower quality class, reinforced with prestressed BFRP fibres.

Type	Specification
NW_BFRP_	non-reinforced solid beams, the European larch *Larix decidua* Mill., originating from an old building erected in 1860, lower quality classes
W_BFRP_—A	solid beams, the European larch *Larix decidua* Mill., originating from an old building erected in 1860, lower quality classes, reinforced with prestressed BFRP fibres, 10 mm in diameter, reinforcement ratio 1.17%

**Table 3:** The results of deflections u (mm) for a series of wooden beam.

BEAM	F/2 (kN)	u (mm)
NW_BFRP_-1	5	17.67
NW_BFRP_-2	5	18.41
NW_BFRP_-3	5	17.51
**Average**		**17.86**
Standard Deviation		0.48
W_BFRP_-A1	5	17.47
W_BFRP_-A2	5	14.19
W_BFRP_-A3	5	13.80
**Average**		**15.15**
Standard deviation		2.02

**Table 4:** The results of tensile and compressive stresses for a series of wooden beams.

Beam	F/2 (kN)	Tensile Stress of Wood (MPa)	Compressive Stress of Wood (MPa)	Tensile Stresses of BFRP Fibres (MPa)
NW_BFRP_-1	5	16.30	−13.73	-
NW_BFRP_-2	5	17.14	−14.35	-
NW_BFRP_-3	5	10.38	−14.07	-
**Average**	**5**	**14.61**	**−14.05**	-
Standard Deviation	-	3.68	0.31	-
W_BFRP_-A1	5	21.94	−12.51	83.61
W_BFRP_-A2	5	15.52	−13.73	53.75
W_BFRP_-A3	5	13.96	−15.97	48.21
**Average**	**5**	**17.14**	**−14.07**	**61.86**
Standard Deviation	-	4.23	1.75	19.04

## References

[1] Yusof A., Saleh A.L. (2010). Flexural Strengthening of Timber Beams Using Glass Fibre Reinforced Polymer. Electron. J. Struct. Eng..

[2] Buell T.W., Saadatmanesh H. (2005). Strengthening Timber Bridge Beams Using Carbon Fibre. J. Struct. Eng..

[3] Valdes M., Giaccu G.F., Meloni D., Concu G. (2020). Reinforcement of maritime pine cross-laminated timber panels by means of natural flax fibers. Constr. Build. Mater..

[4] Wei Y., Ji X., Duan M., Li G. (2017). Flexural performance of bamboo scrimber beams strengthened with fiber-reinforced polymer. Constr. Build. Mater..

[5] Chun Q., Balen K.V., Pan J.W. (2014). Experimental study on flexural performance of small fir and pine timber beams strengthened with near-surface mounted carbon-fiber-reinforced polymer plates and rods. Int. J. Archit. Herit..

[6] Glišović I., Stevanović B., Petrović M. (2015). Bending behaviour of glulam beams reinforced with carbon FRP plates. J. Civ. Eng. Manag..

[7] Güçhan N.S. (2007). Observations on earthquake resistance of traditional timberframed houses in Turkey. Build. Environ..

[8] Brol J., Wdowiak A. (2017). The Use of Glass and Aramid Fibres for the Strengthening of Timber Structures.

[9] Brol J., Nowak T., Wdowiak A. (2018). Numerical Analysis and Modelling of Timber Elements Strengthened with FRP Materials.

[10] Wdowiak A. (2015). Assessment of technical condition of wooden structures. Proc. Transcom.

[11] Wdowiak A. (2016). Analysis of bent timber beam reinforcement with the application of composite materials. Struct. Environ..

[12] Wdowiak A. (2019). Structural and strength properties of bended wooden beams reinforced with fiber composites. Ph.D. Thesis.

[13] Wdowiak A., Brol J. (2019). Effectiveness of Reinforcing Bent Non-Uniform Pre-Stressed Glulam Beams with Basalt Fibre Reinforced Polymers Rods. Materials.

[14] Brol J., Wdowiak-Postulak A. (2019). Old Timber Reinforcement with FRP. Materials.

[15] Wdowiak A., Kroner A. (2017). Influence of the heterogeneity of the structure of bent glued laminated beams on the strengthening effect. Mater. Bud..

[16] Rudzinski L., Wdowiak A. (2016). Strength and structural properties of structural timber. Struct. Environ..

[17] Wdowiak A. (2016). Using the visual method to sort Polish pine structural sawn timber with respect to strength. Czas. Tech..

[18] Wdowiak A. (2017). Defects in structural timber. Struct. Environ..

[19] Wdowiak A., Brol J. (2019). Methods of strength grading of structural timber–comparative analysis of visual and machine grading on the example of Scots pine timber from four natural forest regions of Poland. Struct. Environ..

[20] Wdowiak A. (2017). Examination of structural and geometric features during the strength sorting of construction timber using the visual method. Prz. Bud..

[21] Wdowiak A. (2017). Structure of construction timber. J. Civ. Eng. Environ. Archit..

[22] Li Y.F., Xie Y.M., Tsai M.J. (2009). Enhancement of the flexural performance of retrofitted wood beams using CFRP composite sheets. Constr. Build. Mater..

[23] Borri A., Corradi M., Grazini A. (2005). A method for flexural reinforcement of old wood beams with CFRP materials. Compos. Part B Eng..

[24] Corradi M., Borri A. (2007). Fir and chestnut timber beams reinforced with GFRP pultruded elements. Compos. Part B Eng..

[25] Plevris N., Triantafillou T.C. (1992). FRP Reinforced Wood as Structural Material. J. Mater. Civ. Eng. ASCE.

[26] Schober K.U., Rautenstrauch K. Experimental investigation on flexural strengthening of timber structures with CFRP. Proceedings of the International Symposium on Bond Behaviour of FRP in Structures (BBFS 2005).

[27] Schober K.U., Rautenstrauch K. (2007). Post-strengthening of timber structures with CFRP’s. Mater. Struct..

[28] Jankowski L.J., Jasieńko J., Nowak T.P. (2010). Experimental assessment of CFRP reinforced wooden beams by 4-point bending tests and photoelastic coating technique. Mater. Struct..

[29] Fiorelli J., Alves Dias A. (2003). Analysis of the strength and stiffness of timber beams reinforced with carbon fiber and glass fiber. Mater. Res..

[30] Borri A., Corradi M. (2011). Strengthening of timber beams with high strength steel cords. Compos. Part B.

[31] Gentile C., Svecova D., Saltzberg W., Rizkalla S.H. Flexural strengthening of timber beams using GFRP. Proceedings of the Third International Conference on Advanced Composite Materials for Bridges and Structures.

[32] Alam P., Ansell M.P., Smedley D. (2009). Mechanical Repair of Timber Beams Fractured in Flexure Using Bonded-in Reinforcements. J. Compos. Part B..

[33] Gardner G.P. A Reinforced Glued Laminated Timber System. Proceedings of the Second Pacific Timber Engineering Conference.

[34] Haiman M., Zagar Z. Strengthening the Timber Glulam Beams with FRP Plates. Proceedings of the 7th World Conference on Timber Engineering (WCTE 2002).

[35] (2013). Construction timber graded by strength methods.

[36] (2004). Moisture content of a piece of sawn timber—Part 2: Estimation by electrical resistance method.

[37] (2007). Moisture content of a piece of sawn timber—Part 3: Estimation by capacitance method.

[38] (2012). Timber structures—Structural timber and glued laminated timber—Determination of some physical and mechanical properties.

